# The Mediating Role of Dispositional Optimism and Perfectionism on the Relationship Between Perceived Parental Psychological Control and Support and Adolescents’ Well-Being

**DOI:** 10.3390/ejihpe15080160

**Published:** 2025-08-14

**Authors:** Luana Sorrenti, Maria Imbesi, Carmelo Francesco Meduri, Angelo Fumia, Pina Filippello

**Affiliations:** 1 Department of Clinical and Experimental Medicine, University of Messina, 98124 Messina, Italy; luana.sorrenti@unime.it (L.S.); giuseppa.filippello@unime.it (P.F.); 2 Department of Health Sciences, University Magna Graecia of Catanzaro, 88100 Catanzaro, Italy; maria.imbesi@studenti.unicz.it (M.I.); carmelofrancesco.meduri@unicz.it (C.F.M.)

**Keywords:** well-being, perfectionism, adolescence, optimism, parental control, parental support

## Abstract

Adolescents’ psychological well-being results from the interaction between individual traits, such as optimism and perfectionism, and contextual factors. According to the Self-Determination Theory (SDT), the living environment can promote well-being by fulfilling basic psychological needs. Perceived parental support or control may influence the satisfaction of these needs and the development of dispositional traits, with significant consequences on well-being. This study, conducted on a sample of Italian adolescents (N = 500, M_age_ = 18; SD = 0.7), aimed to explore the mediating role of dispositional optimism and both adaptive and maladaptive perfectionism in the relationship between perceived parental support and control and adolescents’ well-being. Structural Equation Model (SEM) results showed that optimism mediated the relationship between paternal support and well-being (β = 0.029, *p* = 0.05), while adaptive perfectionism mediated the effects of both maternal and paternal support on well-being (β = 0.062, *p* < 0.001; β = 0.038, *p* = 0.001). In contrast, maternal control had an indirect negative impact on well-being through dispositional optimism and maladaptive perfectionism (β = −0.045, *p* = 0.012; β = −0.040, *p* = 0.009), whereas paternal control was not significant. These findings underscore the importance of supportive parenting in promoting adolescent psychological well-being and the risks associated with excessive control.

## 1. Introduction

The World Health Organization defines well-being as a positive state experienced by individuals, embracing quality of life and the capacity to contribute to the world with a sense of meaning and purpose (WHO, 2021). This conceptualization aligns with Ryan and Deci’s (2001) framework of well-being, defined as a multidimensional phenomenon composed of hedonic and eudaimonic perspectives. The hedonic approach considers well-being as a pursuit of pleasure, life satisfaction, and low levels of negative affect, while eudaimonic perspective is characterized by a sense of purpose, self-determination, and the ability to build positive relationships (Stewart-Brown, 2017; Ryff, 1989; Waterman, 1993; Suh et al., 2017). These two perspectives, if combined, best describe the complex construct of psychological well-being (Rose et al., 2017).

This concept has been embraced by Positive Psychology, which shifted the scientific focus from pathology to the study of well-being, emphasizing personal strengths, environmental resources and the conditions that enable individuals and communities to achieve well-being (Viejo et al., 2018). Seligman (2011), a leading figure of Positive Psychology, conceptualizes well-being as the complete realization of an individual’s potential and optimal functioning, a state he defines with the term *flourishing*. Seligman’s theory finds practical application through the PERMA model, in which he theorized the existence of five interdependent dimensions, whose combination leads to psychological well-being: Positive Emotions (P), Engagement (E), Relationships (R), Meaning (M), and Accomplishments (A). According to this model, daily cultivation of these five dimensions allows to reach flourishing (Seligman, 2011; Giangrasso, 2021).

This theorization of well-being is particularly significant if applied to adolescence. Being a developmental period full of physical, psychological, and social changes, adolescence makes individuals vulnerable to a range of adversities (Moreira et al., 2015; Viejo et al., 2018; Sorrenti et al., 2025a), since they often have to face different stressors, whose impact can significantly affect perceived well-being (Madan & Rastogi, 2023). Furthermore, adolescence represents a crucial moment in which many of the factors that contribute to lifelong well-being are either acquired and solidified or not (Ross et al., 2020; Richard & Huppert, 2010; Rathi & Rastogi, 2007). For these reasons, it is important to focus research attention on adolescents’ well-being and how to address their psychological needs.

There are various key factors, both individual and contextual, that may have an impact on adolescents’ psychological well-being. As regards psychological variables, dispositional optimism and perfectionism have been shown to be prominent factors affecting adolescents’ well-being (Suh et al., 2017; Carver & Scheier, 2024). Dispositional optimism is a rather stable dimension of personality, characterized by the expectation that good things will happen and an active and proactive attitude towards adversities (Segerstrom et al., 2017; Carver & Scheier, 2024). Many studies have associated dispositional optimism with physical and psychological well-being, since optimistic individuals seem to be more aware of the importance of physical health, adopt healthier behaviors and adaptive coping strategies, and are more engaged in social relationships than pessimistic individuals (Marrero Quevedo & Carballeira Abella, 2010; Ang, 2024; Buzzai et al., 2020). Research focused on adolescents’ well-being has shown that dispositional optimism is related to subjective well-being (SWB) (Diener et al., 2018; Wrosch et al., 2025) and that optimistic adolescents, who have positive expectations for the future, show higher levels of psychological well-being (PWB) (Oriol & Miranda, 2024). Moreover, a study conducted by Ben-Zur (2003) showed that optimism may contribute to adolescents’ well-being by buffering the effects of stress as well as by promoting active coping and engagement in healthy behaviors.

Perfectionism is a psychological construct characterized by the motivation to pursue excellence (Stoeber, 2017). Perfectionist individuals usually adopt a cognitive style based on a dichotomous all-or-nothing mindset and set extremely high standards, often accompanied by critical evaluations of their own performance (Kamushadze et al., 2021). Stoeber and Otto (2006) highlighted the multidimensional nature of perfectionism and distinguished two major facets: adaptive and maladaptive. Adaptive perfectionism has been described as positive perfectionism, since it is characterized by striving for high standards, organization, the tendency of getting pleasure from performance without fear of evaluation, and the development of adaptive coping strategies (Kamushadze et al., 2021; Suh et al., 2017). On the other hand, the negative dimension of perfectionism is represented by negative attitudes towards mistakes and a feeling of discrepancy between performance and expectations. This maladaptive aspect has been related to negative psychological functioning, such as depressive and anxiety disorders (Stoeber & Otto, 2006; Stoeber & Rambow, 2007). Maladaptive perfectionists tend to be more self-critical, more hesitant about their performance, and more likely to adopt dysfunctional coping strategies, such as avoidance (Li et al., 2015). Research has begun to highlight the impact that perfectionism may have on mental and physical health, as well as well-being (Sirois & Molnar, 2016; Limburg et al., 2017). In recent years, maladaptive perfectionism has increased among young people, who also report higher levels of fatigue, anxiety, depression, and hostility (Fernández-García et al., 2022). While setting high standards may have positive consequences, such as higher motivation and achievement, self-criticism may reduce well-being (Stoeber & Otto, 2006). Adaptive perfectionism is generally associated with positive outcomes, including greater meaning in life, subjective happiness, and life satisfaction, and lower levels of psychological disorders and social difficulties. In contrast, individuals with high levels of maladaptive perfectionism exhibit an intense search for meaning (Suh et al., 2017), increased psychological distress, emotion dysregulation, and low self-compassion (Kamushadze et al., 2021). Thus, psychological functioning appears to vary according to different perfectionistic tendencies.

Considering contextual variables, the idea that social background can promote individual well-being is in line with Self-Determination Theory, an approach proposed by Ryan and Deci (2000) focused on the social-contextual conditions that facilitate or forestall healthy psychological development and well-being. Their findings led to the differentiation of three basic psychological needs: *competence*, the capacity to be able and confident to reach desired objectives; *autonomy*, the ability to make decisions and take initiatives in accordance with personal values; and *relatedness*, based on the sense of feeling connected to one’s own life context and establishing positive and secure relationships with others. The authors conceptualized social environments according to these needs: those that foster autonomy versus those that are controlling and demanding, those that support individuals versus those that are excessively challenging or undermining, and those that are relationally supportive versus those that are rejecting. If social contexts are responsive to these psychological needs, they contribute to increasing levels of well-being by providing constructive feedback and fostering self-regulation, caring, and responsive interpersonal connections. On the other hand, excessive control, nonoptimal challenges, and lack of connectedness can affect well-being and result not only in a lack of initiative and responsibility but also in distress and psychopathology (Ryan & Deci, 2017). Many studies have confirmed these findings, showing that individuals with high levels of social support have higher levels of well-being and fewer emotional and behavioral difficulties.

In particular, parents appear to have a strong effect on self-worth, psychological and physical well-being, by supporting or thwarting these fundamental needs (Hoferichter et al., 2021). At the same time, peer relationships also play a vital role in adolescent life, often becoming the main source of emotional support and showing strong associations with psychological, social, and academic outcomes (Madan & Rastogi, 2023; Sorrenti et al., 2024). However, parents continue to exert a significant influence on adolescents’ psychological adjustment and overall development (Badenes-Ribera et al., 2019; Sechi et al., 2020; Fabris et al., 2025).

Adolescence is a period in which individuals begin to seek their independence and autonomy, experiencing changes in roles and status that may lead to conflicts with parents. This process of individualization can unfold in a warm and open context or, on the contrary, in a hostile and alienated environment, with consequences on adolescents’ well-being and adjustment. Adolescents reporting warm relationships and open communication with their parents show higher levels of well-being and internal resources, such as resiliency, and lower levels of depressive symptoms (Ben-Zur, 2003). Moreover, more perceived support and lower levels of conflict are associated with lower levels of negative affect (İ. B. Arslan et al., 2024) and adolescents’ positive self-evaluations (King et al., 2024). A perceived lack of parental support, especially if associated with the presence of parental psychological control, is related to a greater presence of internalized and externalized problems (Costa et al., 2015), distress, and impaired psychological functioning (Inguglia et al., 2018; G. Arslan, 2018). Parental psychological control is a form of influence enacted by parents using intrusive and manipulative strategies, such as isolation, inducing guilt, and withdrawal of care, to make children modify their opinions, emotions, and behaviors (Barber, 1996). Parental psychological control may induce internalizing symptoms (Costa et al., 2019a) and more antisocial behaviors (Ozdemir, 2012), adversely affecting adolescent adjustment and well-being (X. Yu et al., 2021). These effects can be exacerbated by parental rejection, which has been shown to predict higher levels of psychological distress in adolescents (Fabris et al., 2025).

In the Italian cultural context, parents–adolescent relationships are marked by emotional closeness, high parental involvement, and prolonged co-residence, reflecting the Mediterranean model of “familism” (Crocetti & Meeus, 2014). Italian adolescents often perceive their parents as warm and communicative, but also more behaviorally and psychologically controlling than peers of other Western countries (Claes et al., 2011). Recent studies show that when high parental control is paired with low adolescent frustration tolerance and parents’ own emotion regulation difficulties, it predicts elevated emotion dysregulation and poorer psychological adjustment in youth (Bacchini et al., 2024; Costa et al., 2025; Sorrenti et al., 2015). Furthermore, emerging evidence indicates that lower perceived parental autonomy support alongside higher psychological control is linked to negative emotional and developmental outcomes in Italian adolescents (Papa et al., 2024).

Perceived parental support and control seem to shape more than adolescents’ immediate well-being; they also appear to have an impact on dispositional attributes that carry long-term implications. A family environment characterized by warmth, responsiveness, and open communication is linked to greater levels of dispositional optimism and adaptive forms of perfectionism, qualities associated with superior psychological functioning and social adjustment (DiPrima et al., 2011; Sumer et al., 2009). In contrast, critical or over-controlling parenting predicts lower optimism and promotes maladaptive perfectionism, which, in turn, heightens vulnerability to internalized disorders (Sumer et al., 2009; Enns et al., 2002). Moreover, previous studies have suggested the transdiagnostic nature of perfectionism and supported the transgenerational transmission of perfectionistic traits (Martucci et al., 2023). Exploring these dynamics during adolescence is particularly important, as this developmental stage represents a critical period in which personality traits and emotion regulation strategies are consolidated, potentially shaping long-term mental health trajectories.

### The Present Study

Previous literature has highlighted the role of both individual and social factors in determining different levels of psychological well-being. Positive Psychology recognizes individual traits, such as dispositional optimism and perfectionism, as prominent psychological variables impacting the perceived sense of well-being (Alarcon et al., 2013; Suh et al., 2017). At the same time, other studies, including SDT’s framework (Ryan & Deci, 2017) analyzed the important role of contextual factors, especially perceived parental control and support, in fostering or hindering adolescents’ psychological needs, and consequently well-being (Barber et al., 2005; Leung & Shek, 2020). Perceived parental support and control can also influence adolescents’ individual traits. As outlined above, supportive parenting seems to foster dispositional optimism and adaptive forms of perfectionism (DiPrima et al., 2011; Sumer et al., 2009), while controlling behaviors can lead to maladaptive perfectionism and lower optimism (Sumer et al., 2009; Enns et al., 2002). However, to our knowledge, no study has examined the joint contribution of all these variables to psychological well-being. Considering these theoretical frameworks and adopting a eudaimonic perspective, which encompasses both personal traits and environmental conditions, this study aims to explore the potential mediating role of dispositional optimism and perfectionism, both adaptive and maladaptive, in the relationship between perceived parental support and control (independent variables) and adolescents’ perceived well-being (dependent variable). We expect that perceived parental support will be positively linked to adolescents’ well-being, through higher levels of dispositional optimism and adaptive forms of perfectionism (high standards and organization). Conversely, perceived parental control is expected to show a negative association with well-being, increased levels of maladaptive perfectionism (i.e., discrepancy). Dispositional optimism and the three perfectionism dimensions are expected to function as mediators, helping to clarify how parenting may influence adolescents’ psychological well-being.

## 2. Materials and Methods

### 2.1. Participants

The study sample consisted of 500 adolescents, comprising 270 females (53%), 220 males (45%), and 10 adolescents who chose not to specify their gender (2%). Participants had a range age from 17 to 21 years old (M_age_ = 18; SD_age_ = 0.7) and were all students recruited from high schools in Southern Italy, specifically the Calabria region, that provided their availability.

### 2.2. Instruments

A demographic questionnaire, including age, gender, and school of origin, was used to collect participants’ basic demographic information, while different psychological instruments were administered to assess the variables under study. *The Italian version of the Perma-Profiler* (Giangrasso, 2021) was used to assess adolescents’ general well-being, dependent variable of the study. The questionnaire consists of 23 items, including 15 items related to the five dimensions of Seligman’s model (positive emotions, engagement, relationships, meaning, and accomplishment), 3 items for negative emotions (e.g., “*In general, how often do you feel angry?*”), 3 items for self-perceived physical health (e.g., “*How satisfied are you with your current physical health?*”) and a single item each to assess overall happiness and loneliness (respectively, “*Taking all things together, how happy would you say you are?*” and “*How lonely do you feel in your daily life?*”). All items are rated on an 11-point Likert response scale (0 = not at all, 10 = completely; score range 0–230), with higher scores corresponding to a greater presence of the investigated dimension. However, for the purposes of this study, only the overall well-being score was considered. This score results from the sum of the five PERMA dimensions plus the Happiness scale, yielding a total score ranging from 0 to 160. The PERMA-profiler has demonstrated acceptable reliability and construct validity in previous studies (Butler & Kern, 2016; Buzzai et al., 2020; Giangrasso, 2021).

*The Life Orientation Test-Revised* (Scheier & Carver, 1985) was used to evaluate dispositional optimism, a mediator variable. The instrument is composed of 10 items (e.g., “Overall, I expect more good things to happen to me than bad”, “In uncertain times, I usually expect the best”, “I’m always optimistic about my future”). Each item is assessed on a 5-point Likert-type scale, ranging from 0 (strongly disagree) to 4 (strongly agree). The total score is considered as an overall indicator of dispositional optimism, with higher scores interpreted as reflecting greater levels of optimism. The possible range of scores spans from 0 to 24, since items 2, 5, 6, and 8 serve as fillers and are therefore excluded from the scoring. *The Italian version of the LOT-R* has demonstrated acceptable reliability and construct validity in previous studies (Giannini et al., 2008).

The tendency towards adaptive or maladaptive perfectionism, the other mediator variable, was assessed using *the Italian version of the Almost Perfect Scale-Revised* (Filippello et al., 2016). The instrument consists of 23 items divided into three dimensions: High standards (7 items, e.g., “I have high performance standards at work or school.”; score range 7–49) and Organization (4 items, e.g., “I like to always be organized and precise.”; score range 4–28) reflecting the positive or adaptive aspects of perfectionism, such as the desire to achieve ambitious goals by carefully organizing the necessary actions, and Discrepancy (12 items, e.g., “Doing my best doesn’t seem to be enough for me.”; score range 12–84) representing the maladaptive dimension regarding the perceived gap between self-imposed standards and their actual performance. Responses are evaluated on a 7-point Likert-type scale ranging from 1 (“Totally disagree”) to 7 (“Totally agree”). The reliability and validity of *the Almost Perfect Scale-Revised* have been demonstrated in previous studies (Slaney et al., 2001).

Participants’ perception of parental support, independent variables of this study, was evaluated using the Italian adaptation of the *Perceptions of Parents Scale (POPS)* (Grolnick et al., 1991; Robbins, 1994), which consists of 12 items, assessing mothers and fathers separately with 6 items each. The items are rated on a 7-point Likert scale (1 = Not at all true, 7 = Completely true), with a score ranging from 7 to 42 for each subscale.

Perceived Parental psychological control, the other two independent variables, was assessed using the *Parental Psychological Control Scale* (PCS) (Barber, 1996), composed of 16 items (e.g., “My parents are always trying to change how I feel or think about things’’), evaluating mothers and fathers separately with 8 items each. As POPS, the response system is a 7-point Likert scale ranging from 1 (not at all true) to 7 (very true), with a score ranging from 8 to 56 for each subscale. Both scales are widely used and their psychometric characteristics have been well documented in previous studies (Alivernini & Lucidi, 2011; Costa et al., 2019b; Costa et al., 2025; Cuzzocrea et al., 2020).

For all instruments, raw mean scores were computed and used in the statistical analyses to preserve the original interpretability of the scales and in line with standard practices in educational and psychological research (Boone et al., 2014).

### 2.3. Procedure

This study was performed following the recommendations of the Ethical Code of the Italian Association of Psychology (AIP), and all subjects received written informed consent following the Declaration of Helsinki (2013). The protocol was approved by the Ethics Committee of the Centre for Research and Psychological Intervention (CERIP) of the University of Messina (protocol number: 30465). A total of 12 public high schools across the provinces of the Calabria region were contacted. Of these, 9 schools agreed to participate, while 3 declined due to internal organizational reasons or overlap with other school projects. Recruitment was carried out through direct contact between the research team and school principals, who then involved designated teachers in distributing project information to students. No compensation or incentive of any kind was offered to students or participating schools. Participation was entirely voluntary.

After obtaining ethical approval and the schools’ availability, participants were recruited. The inclusion criteria for participation in the study were as follows: (1) being between 17 and 21 years old, (2) attending a public high school, (3) having a good understanding of the Italian language, and (4) providing signed informed consent from a parent or legal guardian (for minors) or by the student (if of legal age). Students who did not meet these criteria or who did not fully complete the questionnaire were excluded from the study.

The questionnaires were administered from February 2024 to November 2024 through an online survey implemented using Google Forms, in a single period of 20–30 min, during school hours. Students were informed of the project’s aims and given the opportunity to participate voluntarily and anonymously. The research method guaranteed privacy and anonymity of answers and avoided the possibility of receiving incomplete protocols because the online answer insertion did not allow for progress if a response was left unanswered.

### 2.4. Data Analysis

Jamovi 2.6.26 (The Jamovi Project, 2023) was used to conduct descriptive statistics, Cronbach’s alpha, and correlations. A Structural Equation Modeling (SEM) analysis with observed variables was conducted, employing Jamovi’s Semlj package (Gallucci & Jentschke, 2024). The SEM approach was used since it allows multiple dependent variables to be tested simultaneously and has been demonstrated to be superior to traditional univariate and multivariate approaches (Iacobucci et al., 2007; Kline, 2023). A 95% bias-corrected CI was applied following the recommendations (Wu & Jia, 2013; Preacher & Hayes, 2008). To evaluate the fit of the hypothesized model, the following indices were examined: the Chi-square (χ^2^) value; χ^2^/df, to evaluate how well the model fits the data; the Comparative Fit Index (CFI), which compares the proposed model with a null model assuming independence among variables; the Tucker–Lewis Index (TLI), which assesses the fit of the specified model to that of a null model while penalizing for model complexity; Standardized Root Mean Square Residual (SRMR), which measures the discrepancy between observed and predicted correlations; and the Root Mean Square Error of approximation (RMSEA), which measures the discrepancy per degree of freedom in the model. The cut-off for a good model fit is achieved when χ^2^ is <3, the CFI and TLI values are >0.90, and the SRMR and RMSEA values are <0.08.

## 3. Results

### 3.1. Internal Reliability

The internal reliability of the instruments used was assessed by calculating Cronbach’s α for each variable under study. As displayed in Table 1, the mean value, standard deviation (SD), and Cronbach’s α coefficients are reported for each variable to provide a comprehensive description of the internal consistency of the respective items. Except for dispositional optimism, which showed a modest adaptation, Cronbach’s α coefficients were all above 0.70, thus confirming the adequate internal homogeneity and consistency of the scales used in the data collection process.

### 3.2. Correlations

The results of the correlational analysis (Table 2) highlight significant relationships between all variables under study. Overall well-being results positively correlated with dispositional optimism (r = 0.494), high standards perfectionism (r = 0.474), organization perfectionism (r = 0.405), maternal support (r = 0.493), and paternal support (r = 0.429). In contrast, it is negatively correlated with discrepancy perfectionism (r = −0.223), maternal control (r = −0.341) and paternal control (r = −0.221). Dispositional optimism, instead, shows positive correlations with high standard perfectionism (r = 0.245), organization perfectionism (r = 0.166), and maternal and paternal support (r = 0.195 and 0.172, respectively), while it results negatively correlated with discrepancy perfectionism (r = −0.457) and parental control (r = −0.162 and −0.090, respectively). High standards perfectionism is positively correlated with both organization perfectionism and discrepancy perfectionism (r = 0.521 and 0.121, respectively), and with maternal and paternal support (r = 0.375 and 0.273, respectively), and negatively correlated with both maternal and paternal control (respectively, r = −0.193 and −0.125). Similarly, organization perfectionism results positively correlated with parental support (r = 0.288; 0.229) and negatively correlated with parental control (r = −0.178; −0.156). In contrast, discrepancy perfectionism is significantly correlated only with maternal and paternal control (respectively, r = 0.254; 0.154). Finally, from the correlational matrix emerges a strong correlation between maternal and paternal control (r = 0.647), as well as between maternal and paternal support (r = 0.580).

### 3.3. Mediation

**Figure 1:**
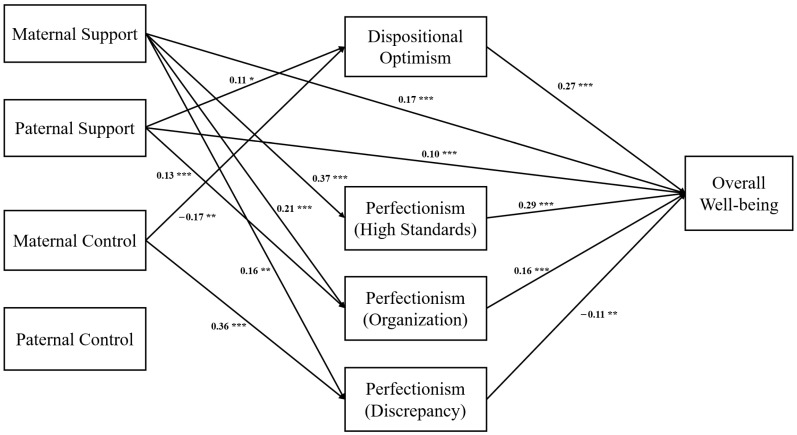
Full mediation model. Note: *** *p* ≤ 0.001, ** *p* ≤ 0.01, and * *p* ≤ 0.05. The coefficients shown are standardized direct path coefficients. The insignificant paths have not been inserted.

Structural Equation Modeling (SEM) was employed to test the hypothesis that dispositional optimism and perfectionism, in its different dimensions (high standards, organization, and discrepancy), could play a role in mediating the relationship between support and control, both maternal and paternal, and adolescents’ overall well-being. The estimation of the model demonstrated a good overall fit to the data [χ^2^ (4) = 7.65, *p* = 0.105, CFI = 0.997, TLI = 0.977, SRMR = 0.021, and RMSEA (90% CI) = 0.043 (0.000, 0.088)], indicating that the proposed structure adequately captured the relationships among the variables. The results (Figure 1) show that overall well-being was positively related to maternal and paternal support (respectively β = 0.17, *p* = ≤0.001 and β = 0.10, *p* = ≤0.001), dispositional optimism (β = 0.27, *p* = ≤0.001), high standards perfectionism (β = 0.29, *p* = ≤0.001), organization perfectionism (β = 0.16, *p* = ≤0.001) and negatively related to discrepancy perfectionism (β = −0.11, *p* = ≤0.01). Dispositional optimism was positively related to paternal support (β = 0.11, *p* = ≤0.05) and negatively related to maternal control (β = −0.17, *p* = ≤0.01). High standards perfectionism was positively related to maternal support (β = 0.37, *p* = ≤0.001). Organization perfectionism, instead, was positively related to both maternal and paternal support (respectively, β = 0.21, *p* = ≤0.001 and β = 0.13, *p* = ≤0.001). Finally, discrepancy perfectionism was positively linked to maternal support and control (respectively, β = 0.16, *p* = ≤0.01 and β = 0.36, *p* = ≤0.001). Regarding the indirect effects of parental support and control on overall well-being, significant indirect effects emerged, (Table 3), as follows: from paternal support to overall well-being via dispositional optimism (β = 0.03, *p* = 0.050, *PM* = 0.18); from maternal control to overall well-being via dispositional optimism (β = −0.05, *p* = 0.012, *PM* = 0.25); from maternal support to overall well-being via organization perfectionism (β = 0.06, *p* = ≤0.001, *PM* = 0.17); from paternal support to overall well-being via organization perfectionism (β = 0.04, *p* = 0.001, *PM* = 0.17); from maternal support to overall well-being via high standards perfectionism (β = 0.06, *p* = ≤0.001, *PM* = 0.31); from maternal support to overall well-being via discrepancy perfectionism (β = −0.02, *p* = 0.049, *PM* = 0.03); and from maternal control to overall well-being via discrepancy perfectionism (β = −0.04, *p* = 0.009, *PM* = 0.12). Moreover, in the present model, the direct path from Maternal control to General well-being was not significant (β = −0.038, *p* = 0.248), indicating that the observed effects operate entirely through the mediators Dispositional optimism and Discrepancy perfectionism. It can also be noted that Paternal control did not show any significant association with the mediating variables or with the outcome variable General well-being. Specifically, the direct path from Paternal control to General well-being was virtually null (β = −0.002, *p* = 0.954). Likewise, all indirect effects through the proposed mediators were non-significant (all *p* > 0.05).

## 4. Discussion

Several studies have highlighted the role of perceived parental support or control, perfectionism, and dispositional optimism on adolescents’ well-being (Kocayörük et al., 2015; Morris & Lomax, 2014; Erickson et al., 2024; Cahill & Gowing, 2024; Oriol & Miranda, 2024). However, to our knowledge, no study has identified the mediating role of dispositional optimism and perfectionism in the relationship between perceived parenting behaviors (supporting or controlling) and adolescents’ overall well-being. Therefore, this study aimed to provide preliminary support for the indirect relationship between perceived parental control and support and adolescents’ well-being through the mediating role of dispositional optimism and perfectionism (high standards, organization, and discrepancy).

Correlational analysis revealed an association between perceived parental support and control with dispositional optimism, all dimensions of perfectionism (high standards, organization, and discrepancy), and adolescents’ overall well-being. Specifically, the results show a positive relationship between well-being and protective factors such as perceived parental support, dispositional optimism, and the adaptive dimensions of perfectionism (high standards and organization), and a negative relationship with risk factors such as discrepancy perfectionism and perceived parental control.

The results obtained from the SEM partially support the research hypotheses. As expected, perceived parental support, dispositional optimism, and adaptive forms of perfectionism (organization and high standards) were positively associated with well-being, whereas perceived maternal control and maladaptive perfectionism (discrepancy) were negatively associated. Contrary to our hypotheses, perceived maternal support was positively associated with discrepancy: this unexpected finding may be due to children’s subjective perception of maternal supportive behaviors. Furthermore, paternal control was not significantly related to any of the examined variables, either directly or indirectly, probably reflecting the gender differences in parenting roles and involvement.

From the analysis of direct relationships, this study shows that perceived maternal support is positively linked to all three dimensions of perfectionism (high standards, organization, and discrepancy), a result consistent with previous literature that showed that parenting styles may be associated with both adaptive and maladaptive forms of perfectionism (Enns et al., 2002). Moreover, this finding aligns with other studies that demonstrated a significant association between perceived maternal support, with maternal encouragement as a moderating factor, and adolescents’ perfectionism, especially when it is perceived as conditional upon performance. In this light, the perceived quality of maternal support (i.e., conditional vs. unconditional) emerges as a critical determinant of adolescents’ internalization of high standards and their self-evaluative processes (Hutchinson & Yates, 2008). Furthermore, the association between perceived maternal support and discrepancy perfectionism may be explained considering adolescents’ subjective perception of parental behaviors (Ingoglia et al., 2021; De Los Reyes et al., 2013; Costa et al., 2025). Thus, maternal supportive and encouraging behaviors may inadvertently be perceived by adolescents as an implicit pressure to meet high standards (Van Petegem et al., 2015), possibly leading to greater perfectionistic discrepancy. Perceived paternal support was positively linked to dispositional optimism. This finding can be explained by considering that perceived paternal support, defined as positive expressiveness and emotional involvement, creates a favorable family environment that may foster the development of children’s dispositional optimism by shaping their perceptions of their own abilities, family relationships, and social surroundings, as described by J. J. Yu and Yeon (2013). Moreover, in our study, perceived paternal support was associated with the adaptive dimension of perfectionism and organization. It has been seen, in fact, that adaptive perfectionists reported significantly higher levels of family support and parental nurturance (LoCicero et al., 2001; DiPrima et al., 2011). A supportive family environment may serve as a safety net for adaptive perfectionists to strive toward their goals, with the perception that they are unconditionally loved, accepted, and supported by their parents, regardless of their performance (DiPrima et al., 2011). Furthermore, our study found that both perceived maternal and paternal support were positively associated with overall well-being. These findings are consistent with the principles of Self-Determination Theory (SDT) (Ryan & Deci, 2000), which states that a family environment characterized by emotional warmth and cognitive stimulation fosters personal growth and optimal psychological functioning. Previous research has also supported the role of perceived parental support in adolescent well-being, showing that perceived parental warmth and autonomy support contribute positively to adolescents’ daily well-being (Bülow et al., 2022) and that individuals who perceive higher levels of parental support exhibit better psychological adjustment, greater life and relationship satisfaction, increased positive affects, and reduced negative ones (Marrero Quevedo & Carballeira Abella, 2010; Govender & Moodley, 2004). As regards the dimension of perceived parental control, this study shows that maternal control has been found to be negatively associated with dispositional optimism and positively with discrepancy perfectionism. The negative influence of perceived maternal control on adolescents’ dispositional optimism aligns with another study that demonstrated that perceived parental psychological control significantly undermines adolescents’ optimism, indirectly acting through lower core self-evaluations (Huang & Shi, 2022). This supports the view that optimism is shaped not only by individual dispositions but also by relational dynamics, particularly parenting practices that restrict autonomy and emotional expression. Moreover, frequent perceived controlling behaviors from mothers have been linked to elevated levels of perfectionism in adolescents, especially socially prescribed perfectionism. This finding holds particular significance for adolescents’ mental health, as socially prescribed perfectionism may act as a key mechanism through which maternal control increases vulnerability to depressive symptoms (Kenney-Benson & Pomerantz, 2005). In our study, dispositional optimism was positively associated with well-being, a finding supported by previous literature (Gallagher & Lopez, 2009; Marrero Quevedo & Carballeira Abella, 2010; Carver & Scheier, 2024; Seligman, 2011). These studies underscore the beneficial role of optimism in enhancing subjective well-being and promoting mental health outcomes, including reduced anxiety, depression, and emotional difficulties. Moreover, optimistic individuals are more likely to perceive their relationships positively, thereby fostering stronger social bonds that contribute to overall well-being. Expanding on this, Ang (2024) identified mindful self-care as a key mediating factor in the relationship between optimism and well-being. Optimists are more inclined to engage in self-care behaviors that support both emotional and physical health, enhancing school and life satisfaction (Sorrenti et al., 2025b). Collectively, these findings highlight the multifaceted role of optimism, through cognitive, social, and behavioral pathways, in promoting psychological well-being, underscoring its value as both a protective factor and a potential target for psychological interventions. Furthermore, all dimensions of perfectionism were associated with overall well-being. Specifically, high standards and organization, core aspects of adaptive perfectionism, are positively associated with well-being. In contrast, discrepancy, a reflection of maladaptive perfectionism, showed a negative relationship. These findings are consistent with prior research. For instance, it was found that individuals with maladaptive perfectionism tend to experience lower well-being unless this effect is buffered by self-compassion (Benedetto et al., 2024). Similarly, it has been reported that adaptive perfectionists demonstrate higher levels of life satisfaction, happiness, and presence of meaning, while maladaptive perfectionists are more likely to experience psychological distress and an intensified search for meaning (Suh et al., 2017). Stoeber and Rambow (2007) further confirmed these patterns in adolescents, showing that striving for perfection is linked to academic motivation and achievement, whereas negative reactions to imperfection predict depressive symptoms and somatic complaints. Taken together, these findings highlight the importance of distinguishing between the different dimensions of perfectionism when evaluating their impact on psychological well-being.

Regarding indirect effects, our results show that perceived paternal support is positively linked to adolescents’ overall well-being through the mediating role of both dispositional optimism and organization perfectionism. Adolescents who perceive higher levels of paternal support are more likely to develop higher levels of dispositional optimism and an adaptive organization-focused form of perfectionism. These psychological traits, in turn, act as intrapersonal resources that facilitate greater psychological well-being, suggesting a cascading effect whereby supportive paternal involvement fosters adaptive cognitive and behavioral patterns conducive to adolescent psychological well-being (J. J. Yu & Yeon, 2013; Hjelle et al., 1996; Kocayörük et al., 2015). In contrast, perceived maternal support is positively associated with well-being via high standards and organization perfectionism, suggesting that supportive maternal behaviors may foster adaptive perfectionistic traits that enhance psychological functioning (Govender & Moodley, 2004). Conversely, perceived maternal support and control were negatively associated with well-being through discrepancy perfectionism. While maternal support is generally considered a protective factor, its link to increased feelings of discrepancy suggests that even autonomy-supportive behaviors may sometimes be perceived by adolescents as pressuring, particularly when they are interpreted as subtle demands or expectations rather than genuine encouragement (Van Petegem et al., 2015; Costa et al., 2025). Perceived maternal control, instead, being associated with criticism and intrusive parenting, may contribute to the development of discrepancy perfectionism by limiting the child’s sense of autonomy and fostering a fear of failure (Hutchinson & Yates, 2008). This, in turn, can increase self-criticism and reduce psychological well-being (Morris & Lomax, 2014; Kenney-Benson & Pomerantz, 2005; Soenens et al., 2008; Filippello et al., 2016). Perceived maternal control is also negatively linked to well-being through dispositional optimism. This result is consistent with previous studies that showed that the lack of perceived parental support, particularly maternal support, and the presence of controlling psychological behaviors, characterized by intrusiveness or excessive regulation of the child’s behaviors and thoughts (Barber, 1996), may have an impact on the development of dispositional optimism by limiting autonomy and reinforcing a perception of the world as unpredictable or threatening (Leung & Shek, 2020). Indeed, controlling behaviors may undermine well-being not only through overt conflict or pressure but also by constraining the child’s ability to develop a positive mindset (X. Yu et al., 2021).

Compared to our research hypothesis, perceived paternal control did not appear to be significantly related, either directly or indirectly, to any of the examined variables. However, this result aligns with previous literature, indicating that especially perceived maternal control exerts a stronger influence on adolescents’ psychological outcomes and overall well-being than perceived paternal control (Furnham & Cheng, 2000; Kenney-Benson & Pomerantz, 2005; Frost et al., 1991; Stafford et al., 2015). This difference may be attributed to mothers’ more direct involvement in the daily regulation of their children’s behaviors and emotions (Bornstein, 2015), often promoting high standards both in behavior, such as organization, and in values, such as meeting expectations, underscoring the distinct influences of maternal versus paternal parenting styles on adolescents’ psychological outcomes.

### Limitations and Future Directions

Despite the valuable insights provided by the current study, several limitations should be acknowledged. First, the cross-sectional nature of the study precludes the establishment of causal inferences. To address this issue in future research, longitudinal studies are needed to examine the directionality and causal nature of the observed relationships, particularly in relation to the developmental trajectory of perfectionism and optimism across adolescence, and to test the robustness of the mediation model over time. Moreover, even if the sample size was adequate, the convenience sample recruited from schools may limit the generalizability of the findings. Since the sample recruited was from Southern Italy, it may not be representative of the broader adolescent population, as it may reflect specific cultural, socioeconomic, or geographical characteristics, limiting the generalizability of the findings to different contexts. However, despite the disparities between Northern, Central, and Southern Italy, the challenges related to adolescence are universal. Nevertheless, future research should expand the sample size nationwide to confirm the validity of these results in different contexts. Another limitation is the exclusive use of self-report instruments to assess constructs, which may introduce biases related to social desirability and subjective perception. Particularly in adolescent populations, self-reports may not always provide accurate reflections of parenting practices or internal psychological traits. Future studies should consider other methods of data collection in addition to self-reports, such as direct observation. Including parental perspectives could provide a more balanced understanding of family dynamics and strengthen the interpretation of the observed effects. Moreover, the present model did not consider potentially influential moderating variables such as gender, age, the quality of the parent–child relationship, exposure to stressful life events, or school-related factors. In particular, gender was not considered since the focus of the study was on identifying general patterns of association applicable across adolescents, regardless of gender. Including gender as a factor in the analyses could have unnecessarily complicated the model without contributing to the research objectives. Moreover, in the literature, there are divergent results concerning gender differences (Kenney-Benson & Pomerantz, 2005; Sumer et al., 2009; Morris & Lomax, 2014; Stafford et al., 2015; Van Petegem et al., 2015). However, these variables could affect the strength and direction of the observed relationships and provide a more nuanced understanding of adolescent well-being, so they should be considered in future studies. Furthermore, in this study were observed rather than latent variables in the structural equation modeling (SEM). The exclusive use of observed variables may have led to an underestimation or overestimation of the true associations among constructs, such as perceived parental support and control, optimism, perfectionism, and well-being. Future studies should consider adopting the latent SEM approach using multiple indicators per construct to enhance the robustness and precision of the findings.

Despite these limitations, this study provides new insights into the complex mechanisms linking parenting styles to adolescents’ psychological well-being, highlighting the mediating roles of dispositional optimism and distinct dimensions of perfectionism. To the best of our knowledge, this is one of the first studies to explore the mediating role of dispositional optimism and perfectionism between these variables among adolescents.

## 5. Conclusions

In conclusion, this study extends the present literature by providing preliminary evidence for the mediating role of internal traits in the parenting–well-being relationship and by supporting the relevance of Self-Determination Theory in understanding how autonomy-supportive versus controlling parenting practices shape the internal resources of adolescents. The findings demonstrate that perceived parental support is positively associated with well-being, both directly and indirectly, through the promotion of optimism and adaptive forms of perfectionism. In contrast, perceived maternal control emerged as a risk factor, negatively impacting well-being via increased maladaptive forms of perfectionism and reduced optimism, emphasizing the importance of considering the differential influence of maternal and paternal figures in adolescents’ psychological development. These findings have several practical implications for parents, educators, and mental health professionals working with adolescents. Interventions aimed at fostering parental emotional warmth and autonomy support, by encouraging open communication, validating adolescents’ feelings, and allowing them to make age-appropriate decisions, can create a more supportive home environment. In contrast, for parents who tend to engage in psychological control, parenting interventions can focus on increasing awareness of controlling behavior and teaching alternative strategies that promote adolescent autonomy, such as motivational interviewing or autonomy-supportive parenting workshops (X. Yu et al., 2021). Furthermore, professionals can help adolescents develop adaptive perfectionistic traits, such as setting realistic goals and learning from mistakes, through goal-setting workshops or structured coaching sessions, while dispositional optimism may be cultivated through cognitive-behavioral techniques that reframe negative thoughts, gratitude exercises, and resilience training programs (Sorrenti et al., 2025b). These strategies may serve as protective factors that enhance psychological resilience and well-being during this critical developmental period.

## Figures and Tables

**Table 1:** Means, SDs, and Cronbach’s α coefficients for each variable analyzed in the study (N = 500).

	Overall Well-Being	Dispositional Optimism	High Standards Perfectionism	Organization Perfectionism	Discrepancy Perfectionism	Maternal Support	Paternal Support	Maternal Control	Paternal Control
**Mean**	7.14	2.02	5.43	5.31	4.43	5.16	4.97	1.64	1.53
**SD**	1.65	0.66	0.96	1.26	1.25	1.52	1.66	0.50	0.51
**α**	0.86	0.65	0.77	0.82	0.89	0.92	0.93	0.84	0.87

**Table 2:** Correlation matrix (N = 500). Note. * *p* < 0.05, ** *p* < 0.01, *** *p* < 0.001.

	1	2	3	4	5	6	7	8	9
1. Overall well-being	—								
2. Dispositional optimism	0.494 ***	—							
3. High standards perfectionism	0.474 ***	0.245 ***	—						
4. Organization perfectionism	0.405 ***	0.166 ***	0.521 ***	—					
5. Discrepancy perfectionism	−0.223 ***	−0.457 ***	0.121 **	0.050	—				
6. Maternal control	−0.341 ***	−0.162 ***	−0.193 ***	−0.178 ***	0.254 ***	—			
7. Paternal control	−0.221 ***	−0.090 *	−0.125 **	−0.156 ***	0.154 ***	0.647 ***	—		
8. Maternal support	0.493 ***	0.195 ***	0.375 ***	0.288 ***	−0.027	−0.555 ***	−0.295 ***	—	
9. Paternal support	0.429 ***	0.172 ***	0.273 ***	0.229 ***	−0.044	−0.335 ***	−0.380 ***	0.580 ***	—

**Table 3:** Path estimates, SEs, 95% CIs and Proportional Mediation (PM).

	*β*	*SE*	*Lower Bound* *(BC)* *95% CI*	*Upper Bound* *(BC)* *95% CI*	*p*	*PM*
***Indirect Effects* via *Dispositional optimism***						
Paternal support → Overall well-being	0.03	0.02	0.00	0.06	0.050	0.18
Maternal control → Overall well-being	−0.05	0.06	−0.27	−0.03	0.012	0.25
***Indirect Effects* via *Organization perfectionism***						
Maternal support → Overall well-being	0.06	0.02	0.05	0.13	<0.001	0.17
Paternal support → Overall well-being	0.04	0.02	0.02	0.08	0.001	0.17
***Indirect Effects* via *High standards perfectionism***						
Maternal support → Overall well-being	0.06	0.01	0.02	0.07	<0.001	0.31
***Indirect Effects* via *Discrepancy perfectionism***						
Maternal support → Overall well-being	−0.02	0.01	−0.04	−0.00	0.045	0.03
Maternal control → Overall well-being	−0.04	0.05	−0.23	−0.03	0.009	0.12

## Data Availability

The data that support the findings of this study are available from the corresponding author upon reasonable request.
